# Digital Nerve Block for the Reduction of a Proximal Phalanx Fracture of the Foot – a Case Report

**DOI:** 10.21980/J8KS8T

**Published:** 2020-01-15

**Authors:** Emerald Raney, John Costumbrado, Barbara Blasko, Dev Dhillon

**Affiliations:** * Riverside Community Hospital/University of California Riverside, Department of Emergency Medicine, Riverside, CA; ^ University of California, Riverside School of Medicine, Riverside, CA

## Abstract

Providing efficient, and patient-centered care is the goal for managing patients in the emergency department (ED). In this case report, we will discuss how performing a digital nerve block was critical in providing analgesia and facilitating the reduction of a proximal phalanx fracture in a timely fashion for a pediatric patient that presented to the ED with a complaint of right toe pain after stubbing her toe. Radiographs showed evidence of an acute, displaced fracture of the 4th phalanx of the right foot. Utilizing the two-sided web space block, the patient was able to tolerate reduction and splinting with buddy taping without complications. The patient remained neurovascularly intact prior to and after the procedure. This case report demonstrates how a digital nerve block can be a safe, effective intervention that can improve ED throughput. The patient’s condition improved throughout her ED course, and she was discharged in stable condition.

**Topics:**

Trauma, fracture, x-ray, digital nerve block.

[Fig f1-jetem-5-1-v25]

**Figure f1-jetem-5-1-v25:**
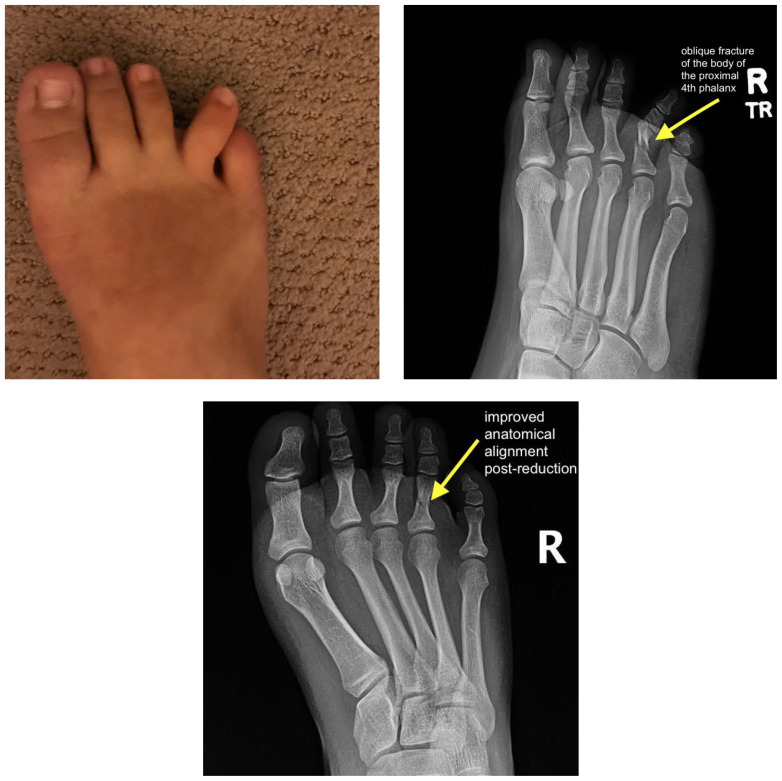


## Introduction

Phalanx fractures are the most common fractures of the foot. A reported 140 cases per 100,000 people per year visit the ED for these injuries.^1^ Most commonly they can be completely managed in the ED without requiring an orthopedic referral. Goals of management include reestablishment of alignment and range of motion, prevention of complications, and pain management.^2^ The most common mechanisms of injury include axial loading and “stubbing” or crush injury.^3^ Regional anesthesia can provide rapid as well as prolonged relief depending upon the agent. Nerve blocks can be useful in the ED setting for more than just temporary analgesia. In this case, we will discuss how a nerve block provided complete anesthesia in the affected digit, which allowed for a more comfortable procedure and culminated in the successful reduction of a proximal phalanx fracture.

## Presenting concerns and clinical findings

The patient is a 15-year-old female with no significant past medical history who presented to the ED with her family for evaluation of right toe pain. She states that she stubbed her toe on a door while running through the house. She describes sharp pain in her right 4th phalanx that worsens with pressure or movement of the toe. Examination of her right foot showed lateral misalignment of the 4th phalanx (see image 1). The patient was neurovascularly intact and had no additional signs of acute traumatic injuries.

## Significant findings

Plain film of the right foot showed evidence of an oblique fracture of the body of the proximal 4th phalanx (image 2). No other acute traumatic injuries noted in the rest of the bones and joints of the right foot. After performing a digital block of the toe and reduction, repeat imaging showed evidence of successful reduction with anatomic alignment and redemonstration of the fracture line (image 3).

## Patient course

The patient presented to the ED with pain to the right foot and an obvious deformity to the right 4th phalanx suggestive of an acute traumatic injury. Differentials were considered. Nonaccidental trauma (NAT) was less likely given the reported mechanism of injury, history, and reassuring observed family dynamics. Pathological fractures were considered less likely given the patient’s unremarkable past medical history and benign exam other than the acute injury. After the diagnosis was confirmed via plain film, the risks, benefits, and alternatives for a digital nerve block and reduction were discussed, and the patient and family opted for the procedure. The two-sided web space block technique was used to facilitate reduction with longitudinal traction. Placing an object (eg, syringe, pen) in the web space can help provide a fulcrum and stability while reducing. In this case a pen was used. Due to the efficacy of the nerve block and fracture reduction, we were able to manage the patient’s injury and discharge the patient from the ED in less than 1 hour. Before leaving the ED, the patient expressed gratitude for the care she received. She revealed that she was initially anxious about undergoing reduction with a digital block; however, the block worked so well that she thought that the mild pain she felt from the administration of the anesthetic agent was more uncomfortable than the reduction and splinting.

## Discussion

The most common mechanisms of phalanx fractures of the foot include axial loading and “stubbing” or crush injury. Patients will typically present with pain with ambulation, tenderness to palpation, swelling and ecchymosis at the affected site and surrounding tissues.^2^,^3^ Initial assessment should include evaluation for nail bed injury and neurovascular compromise. It is important to always compare the affected digit to the other foot in order to help detect deformities. Radiographic evaluation varies depending on the site of injury, but typically should include an anteroposterior, oblique, and lateral view with visualization of the adjacent toes and joints above and below the suspected fracture location.^5^ Passive dorsiflexion of toes 2–5 can help avoid overlap and misidentification of fractures in the smaller digits. Once all injuries are identified, dislocations or misaligned fractures need to be reduced.

Regional anesthesia allows for better pain control during the procedure and can provide rapid as well as prolonged relief depending upon the agent. Most commonly, lidocaine 1% without epinephrine (maximum dose 5 mg/kg) is the agent of choice, which was the case with this patient. Epinephrine is typically avoided in areas such as fingers and toes with small vessels to prevent ischemic complications. Lidocaine has an onset of about two minutes and will last approximately thirty to forty minutes. Bupivacaine 0.25% (maximum dose 2 mg/kg without epinephrine) can last between two to four hours, but has a longer onset for complete anesthesia of about five to ten minutes^4^. Lidocaine and bupivacaine can be combined in a 50:50 ratio typically 5–10mL total to provide immediate and prolonged anesthesia depending on the severity of the injury.^4^,^5^ Nerve blocks can achieve up to complete anesthesia in the affected digit, allowing for a more comfortable procedure and increasing the chance for a successful reduction.

There are multiple techniques for digit blocks. The sensory function of the toes is supplied by 2 dorsal and 2 volar nerves, which branch off the major ankle nerves. All 4 nerves can be blocked due to their sensory overlap.^6^

A common technique, such as with this case, is the two-sided web space block. With the patient’s foot plantar side down, place a small 1-mL skin wheal between the metatarsal bones on the dorsal aspect. Insert the needle at a 90-degree angle on either the medial or lateral aspect just distal to the metatarsal-phalangeal joint. A smaller gauge needle, such as a 27 or 30 gauge, will reduce discomfort with the injection. This technique involves aspirating to ensure the needle tip is not in a vessel and then injecting. Continue to aspirate and inject as the needle is advanced forward. Do not pierce through the skin on the plantar surface. The needle can be directed either medially or laterally depending on the initial insertion site to help ensure adequate anesthesia. Repeat the steps on the opposite side of the digit. Wait 5–10 minutes and then check to see if the block was successful.^5^,^7^,

Another approach is the single injection site technique that is performed similar to the transthecal block of the fingers.^6^ This can be performed by inserting the needle at a 90-degree angle proximal to the metatarsophalangeal joint on the plantar side of the foot until the needle tip hits bone, slightly drawback and aspirate, then inject.^5^ This technique can be more comfortable for patients because it only has one insertion, but may not provide as good anesthesia if the nerve is not completely bathed in the anesthetic. Typically, about 3–5mL of anesthetic will provide sufficient effect.

Reduction is best achieved by moving the displaced bone in the opposite direction of the force involved in causing the injury. During reduction, maintain constant longitudinal traction while manipulating the fragments back into alignment. Increasing the deformity with gentle hyperextension, flexion, or lateral pressure before reducing can help to facilitate reduction.^1^,^2^ Once satisfactory reduction has been achieved, the affected digit can be buddy splinted to the adjacent digit in order to provide support and stability. A hard soled shoe can be used for walking. The patient should be advised to be non-weight bearing and advance as tolerated. A post reduction radiograph should be ordered, and it is also important to always check neurovascular status after reduction.^2^

This case illustrates the point that considering nerve blocks can give ED providers another tool to provide excellent patient care and improve ED throughput by negating the need for IV access, procedural sedation, etc., with a relatively, quick, safe, and effective procedure.

## Supplementary Information










