# Supplementary figures and images for: Digital Nerve Block for the Reduction of a Proximal Phalanx Fracture of the Foot – a Case Report
Source: J Educ Teach Emerg Med. 2020 Jan 15;5(1):V25–8. doi: 10.21980/J8KS8T (PMC10332539; doi:10.21980/J8KS8T)

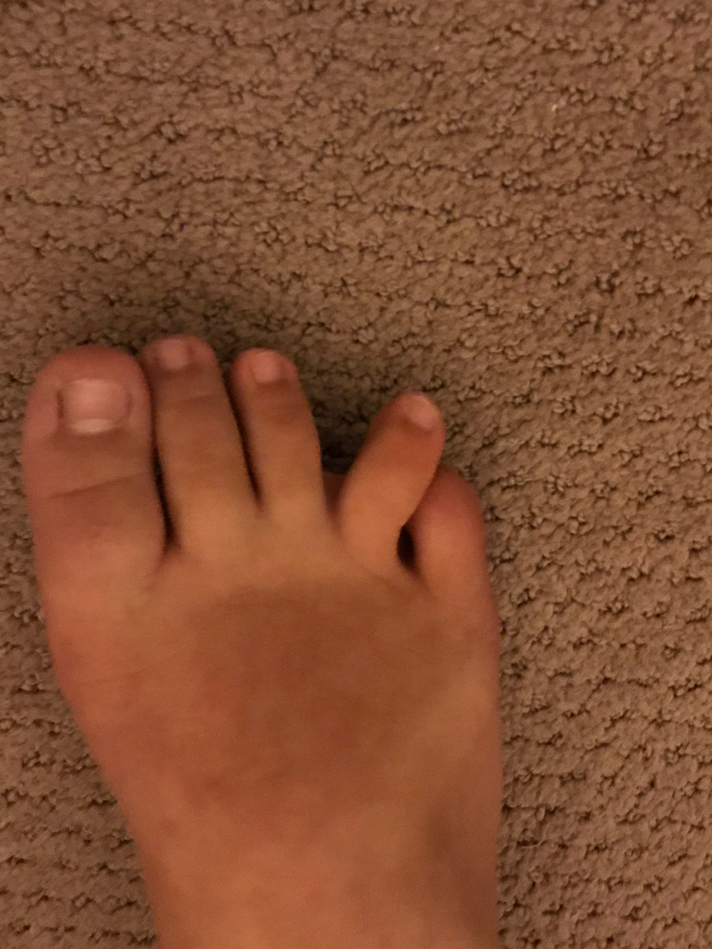

Supplement: Supplementary file 1 [file jetem-5-1-v25-supp1.jpg]

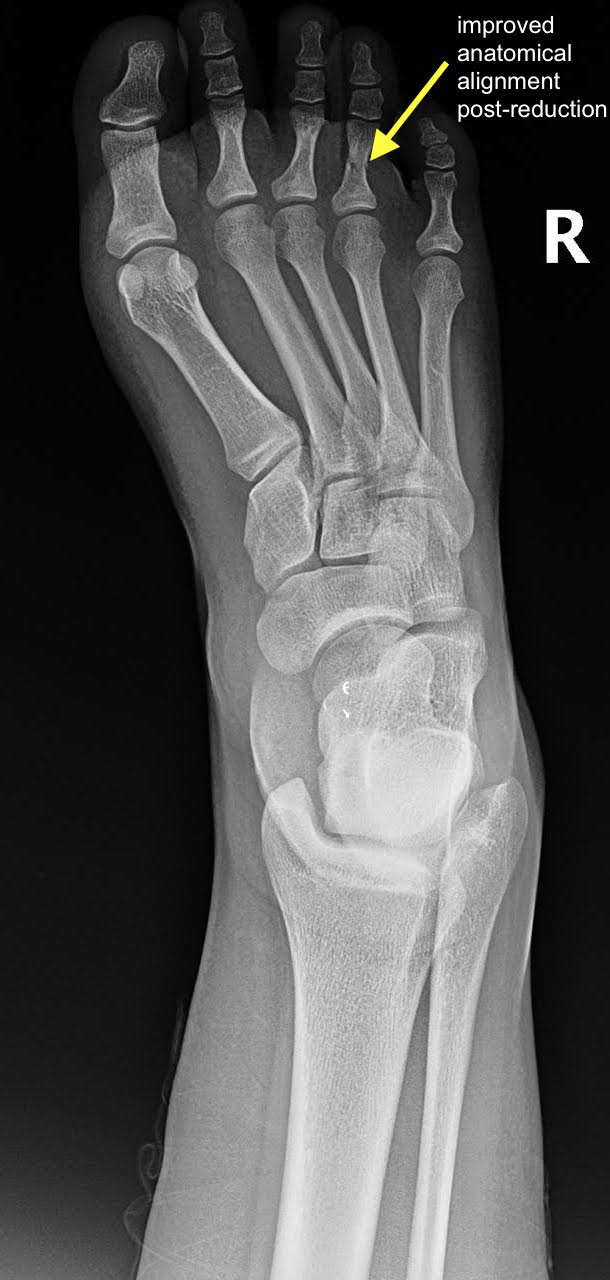

Supplement: Supplementary file 2 [file jetem-5-1-v25-supp2.jpg]

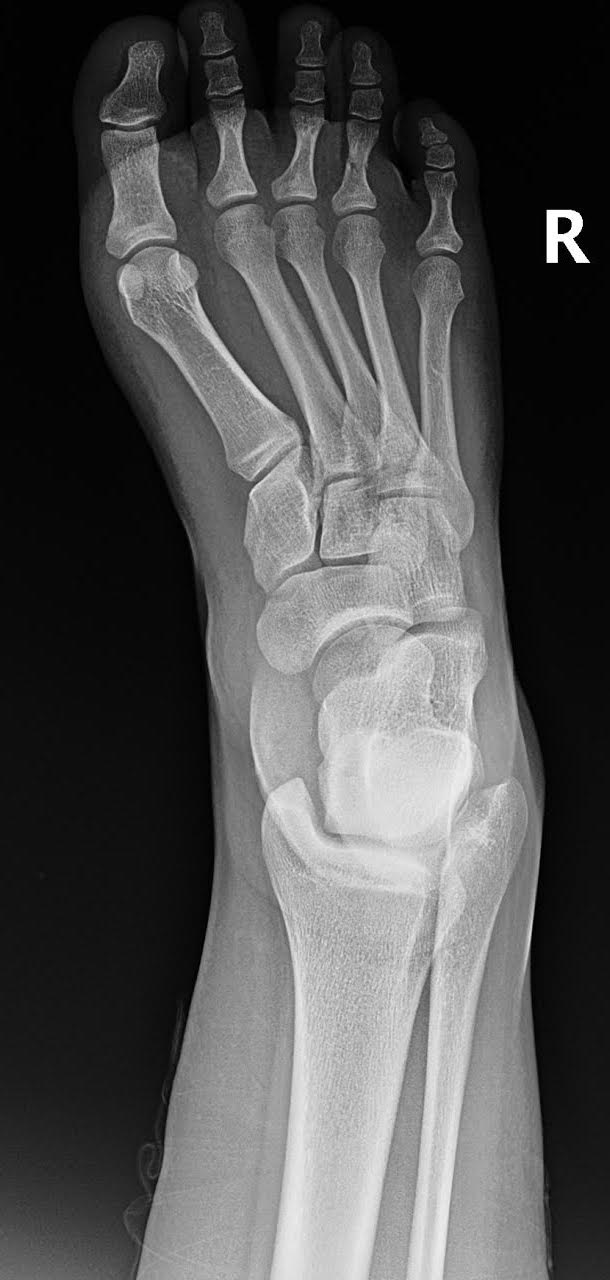

Supplement: Supplementary file 3 [file jetem-5-1-v25-supp3.jpg]

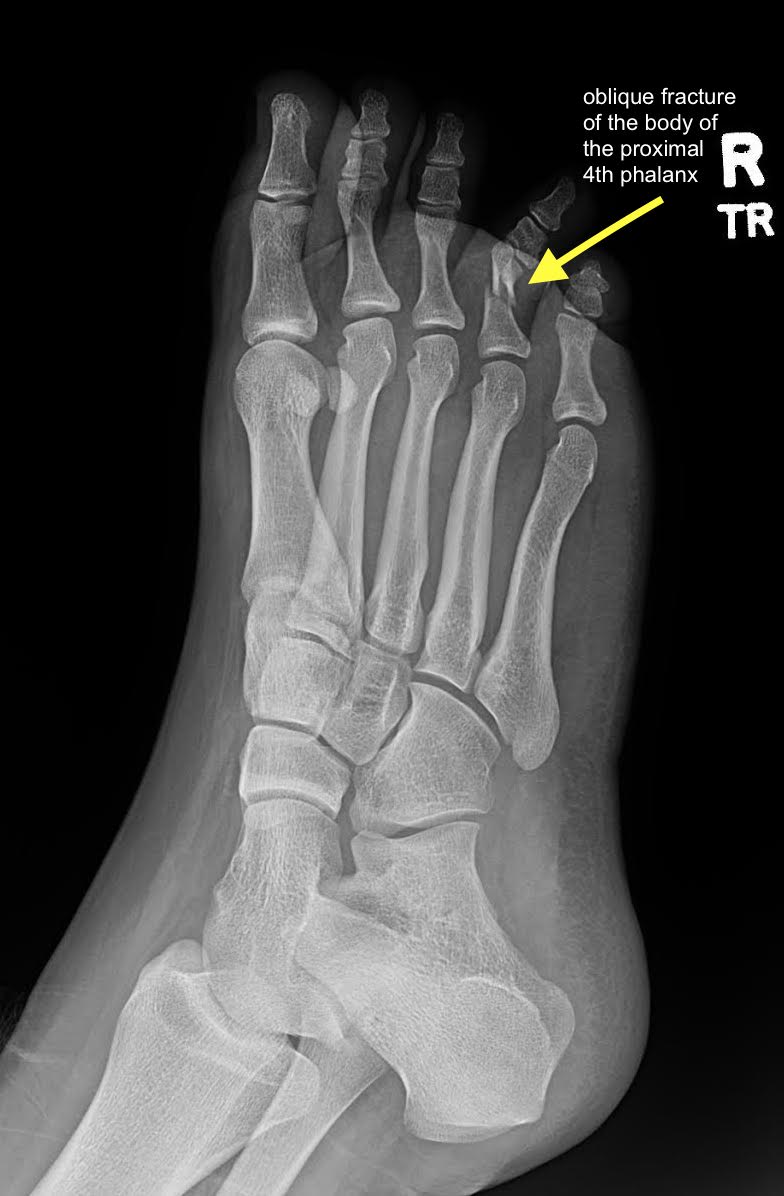

Supplement: Supplementary file 4 [file jetem-5-1-v25-supp4.jpg]

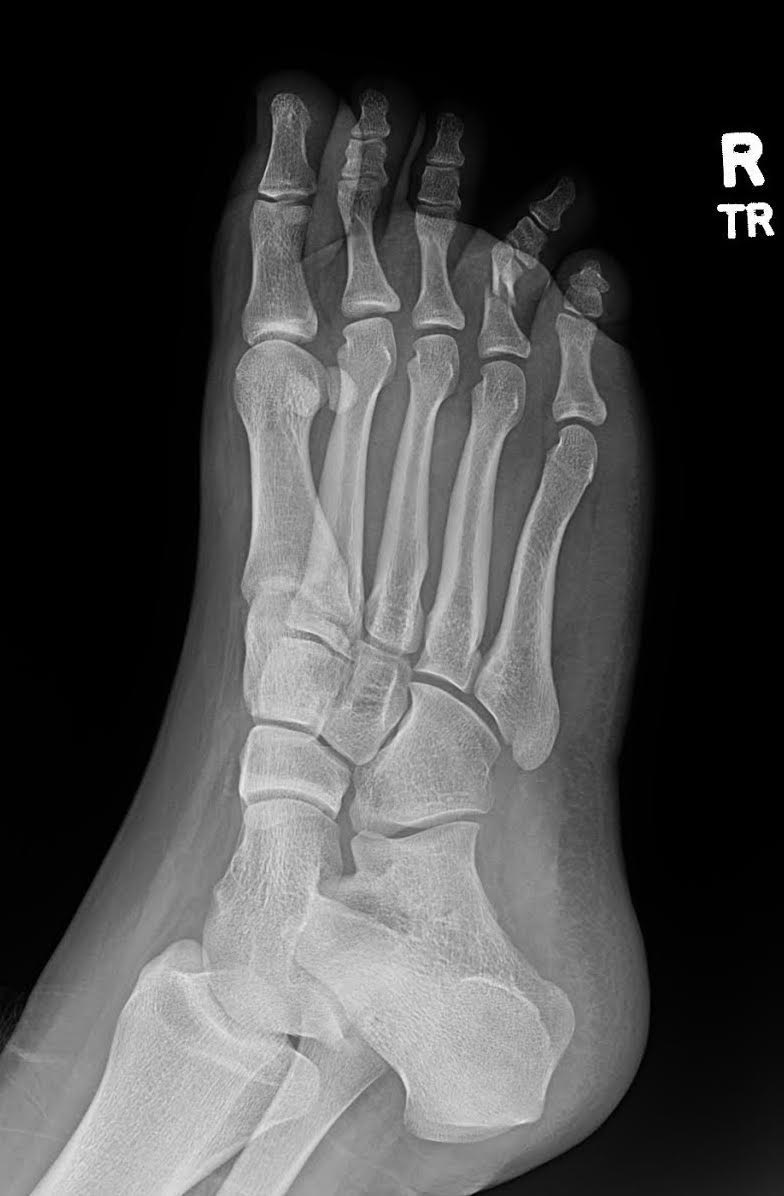

Supplement: Supplementary file 5 [file jetem-5-1-v25-supp5.jpg]
